# Mild Encephalopathy With a Reversible Splenial Lesion Type II in a Probable Post-infectious SARS-CoV-2 Context: A Case Report

**DOI:** 10.7759/cureus.106973

**Published:** 2026-04-13

**Authors:** Abdelhakim Boulfouyoul, Kaoutar Khabbache, Yousra El Boussaadni, Abdallah Oulmaati

**Affiliations:** 1 Department of Pediatrics and Neonatology, The University Hospital Center of Tangier, Tangier, MAR; 2 Department of Pediatrics and Neonatology, Faculty of Medicine and Pharmacy of Tangier, Abdelmalek Essaâdi University, Tangier, MAR

**Keywords:** case report, cerebellar lesions, mers, pediatric encephalopathy, sars-cov-2

## Abstract

Mild encephalopathy with a reversible splenial lesion (MERS) is a clinico-radiological syndrome characterized by acute encephalopathy and a transient lesion in the splenium of the corpus callosum on magnetic resonance imaging (MRI). We report a case of a six-year-old girl who presented with behavioral disturbance and altered mental status after one week of a viral prodrome in the setting of household SARS-CoV-2 exposure. Cerebrospinal fluid analysis was unremarkable, and SARS-CoV-2 serology showed positive immunoglobulin G (IgG) and negative immunoglobulin M (IgM). Brain MRI demonstrated a focal splenial lesion with bilateral cerebellar involvement of the dentate nuclei, consistent with MERS type II. The patient initially received ceftriaxone and acyclovir for suspected encephalitis. Neurological deterioration with dysphagia led to treatment with intravenous methylprednisolone followed by intravenous immunoglobulin. Follow-up MRI at one month showed complete lesion resolution, and complete neurological recovery was achieved by three months. This case highlights the importance of recognizing this reversible clinico-radiological pattern in children with acute encephalopathy to facilitate diagnosis, guide management, and avoid unnecessary investigations.

## Introduction

Mild encephalopathy with a reversible splenial lesion (MERS) is a distinct clinico-radiological syndrome characterized by transient encephalopathy and a reversible lesion of the splenium of the corpus callosum on magnetic resonance imaging (MRI) [1]. These lesions are part of the broader spectrum of cytotoxic lesions of the corpus callosum and occur in infectious, autoimmune, metabolic, seizure-related, and drug-related conditions [2].

Since the COVID-19 pandemic, MERS and related reversible splenial lesion syndromes have been reported in association with SARS-CoV-2, including pediatric cases [3-5]. In children, MERS is classified as type I, limited to the splenium, or type II, with lesions beyond it [6,7]. Extra-splenial lesions may involve the cerebellum [8,9], and type II may recover more slowly than type I [7]. We report a case of MERS type II in a six-year-old girl with bilateral cerebellar lesions involving the dentate nuclei in the setting of probable post-infectious SARS-CoV-2 infection.

## Case presentation

Patient information

In November 2021, a six-year-old girl with previously normal neurodevelopment was admitted for altered mental status and behavioral disturbance. Her routine childhood immunizations were up to date, and she had not received the SARS-CoV-2 vaccination. She was born to non-consanguineous parents. Her past medical history was unremarkable, with no epilepsy, no exposure to or withdrawal of antiepileptic drugs, and no use of drugs such as metronidazole or cyclosporine.

There was a history of household SARS-CoV-2 exposure. Her father had confirmed COVID-19 about one month before presentation, and several other household members later developed symptoms compatible with COVID-19, although they were not tested.

Clinical findings

Symptoms began seven days before admission with a viral prodrome consisting of fever, cough, rhinorrhea, myalgia, vomiting, and headache. On admission, she developed acute behavioral disturbance with agitation and mutism. On examination, her temperature was 37.1°C, her weight was 16 kg, and her capillary blood glucose was 97 mg/dL. She was hemodynamically stable, with no respiratory distress. Neurological examination showed a Glasgow Coma Scale score of 12/15 (E4V2M6), poor eye contact, hypotonia, and marked motor weakness affecting all four limbs. There were no pyramidal signs. Cerebellar testing could not be reliably assessed at admission because of marked psychomotor agitation and hypotonia.

Diagnostic assessment

Lumbar puncture performed on admission showed clear cerebrospinal fluid (CSF) with five white blood cells/mm³ and five red blood cells/mm³, protein 0.17 g/L, and glucose 0.41 g/L. CSF bacterial culture was sterile, and the multiplex CSF polymerase chain reaction (PCR) panel was negative.

Laboratory tests showed an inflammatory response with C-reactive protein of 36.7 mg/L and transient transaminase elevation (aspartate aminotransferase: 222 U/L, alanine aminotransferase: 117 U/L), with improvement three days later (aspartate aminotransferase: 48 U/L, alanine aminotransferase: 41 U/L). The complete blood count was unremarkable. Electrolytes were within normal ranges, notably sodium 136 mmol/L. Renal function was normal (urea: 0.4 g/L, creatinine: 4.5 mg/L). Serum glucose was 0.90 g/L. SARS-CoV-2 serology showed positive immunoglobulin G (IgG) and negative immunoglobulin M (IgM). Laboratory and cerebrospinal fluid findings on admission are summarized in Table 1.

**Table 1:** Laboratory and cerebrospinal fluid findings on admission.

Categories	Parameters	Results	Reference range
Complete blood count	White blood cell count	8,090 cells/µL	4,000-10,000 cells/µL
	Neutrophils	5,100 cells/µL	1,500-7,000 cells/µL
	Lymphocytes	1,840 cells/µL	1,000-4,800 cells/µL
	Hemoglobin	12.3 g/dL	11.5-17.5 g/dL
	Platelets	449,000 cells/µL	150,000-450,000 cells/µL
Inflammatory marker	C-reactive protein	36.7 mg/L	<5 mg/L
Liver enzymes	Aspartate aminotransferase	222 U/L	0-40 U/L
	Alanine aminotransferase	117 U/L	0-40 U/L
Serum biochemistry	Sodium	136 mmol/L	135-145 mmol/L
	Potassium	4.9 mmol/L	3.5-5.0 mmol/L
	Chloride	102 mmol/L	97-111 mmol/L
	Calcium	91 mg/L	90-106 mg/L
	Urea	0.4 g/L	0.11-0.45 g/L
	Creatinine	4.5 mg/L	4.5-8.1 mg/L
	Serum glucose	0.90 g/L	0.7-1.1 g/L
Serology	SARS-CoV-2 immunoglobulin G (IgG)	Positive	-
	SARS-CoV-2 immunoglobulin M (IgM)	Negative	-
Cerebrospinal fluid	Appearance	Clear	Clear
	White blood cells	5 cells/mm³	<10 cells/mm³
	Red blood cells	5 cells/mm³	<10 cells/mm³
	Protein	0.17 g/L	0.15-0.45 g/L
	Glucose	0.41 g/L	0.4-0.8 g/L
	Bacterial culture	Sterile	Sterile
	Multiplex polymerase chain reaction panel	Negative	Negative

**Figure 1:**
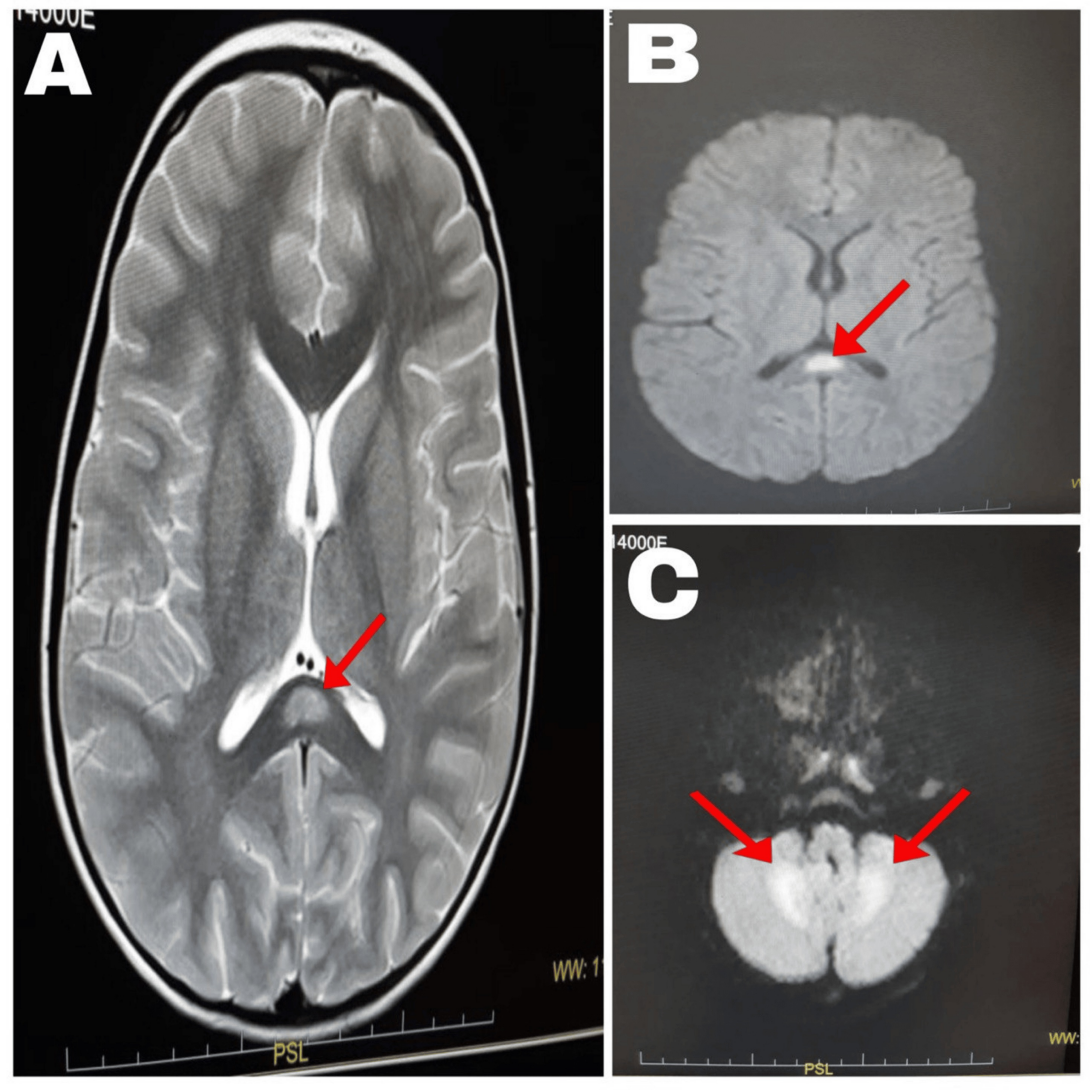
Initial brain magnetic resonance images showing splenial and bilateral cerebellar lesions. (A) Axial T2-weighted image showing a focal hyperintense lesion in the splenium of the corpus callosum (red arrow). (B) Axial fluid-attenuated inversion recovery image confirming the splenial lesion (red arrow). (C) Axial diffusion-weighted image showing bilateral cerebellar lesions involving the dentate nuclei (red arrows).

Brain computed tomography performed before lumbar puncture was normal. Brain MRI on hospital day five showed a focal splenial lesion hyperintense on T2-weighted and fluid-attenuated inversion recovery (FLAIR) sequences, with diffusion restriction and no gadolinium enhancement, along with bilateral cerebellar lesions involving the dentate nuclei with diffusion restriction, consistent with MERS type II (Figures 1A-1C). Electroencephalography was attempted twice but was not completed because of psychomotor agitation.

Therapeutic intervention

Empirical treatment for suspected encephalitis was started on hospital day one with intravenous ceftriaxone 100 mg/kg/day and intravenous acyclovir 20 mg/kg every eight hours, both of which were stopped after the initial infectious workup and brain MRI results became available. Neurological deterioration occurred on hospital day six, with loss of visual contact and dysphagia. After major infectious and metabolic causes had been excluded, immunomodulatory treatment was started for suspected post-infectious immune-mediated encephalopathy within the MERS spectrum. She received intravenous methylprednisolone pulse therapy (30 mg/kg/day) for three days (hospital days seven to nine) without clear early improvement, followed by intravenous immunoglobulin on hospital days 12-13 (1 g/kg/day for two days; total dose 2 g/kg). Motor physiotherapy and speech therapy were initiated during hospitalization and continued after discharge. No adverse events were observed with corticosteroids or intravenous immunoglobulin.

Follow-up and outcomes

**Figure 2:**
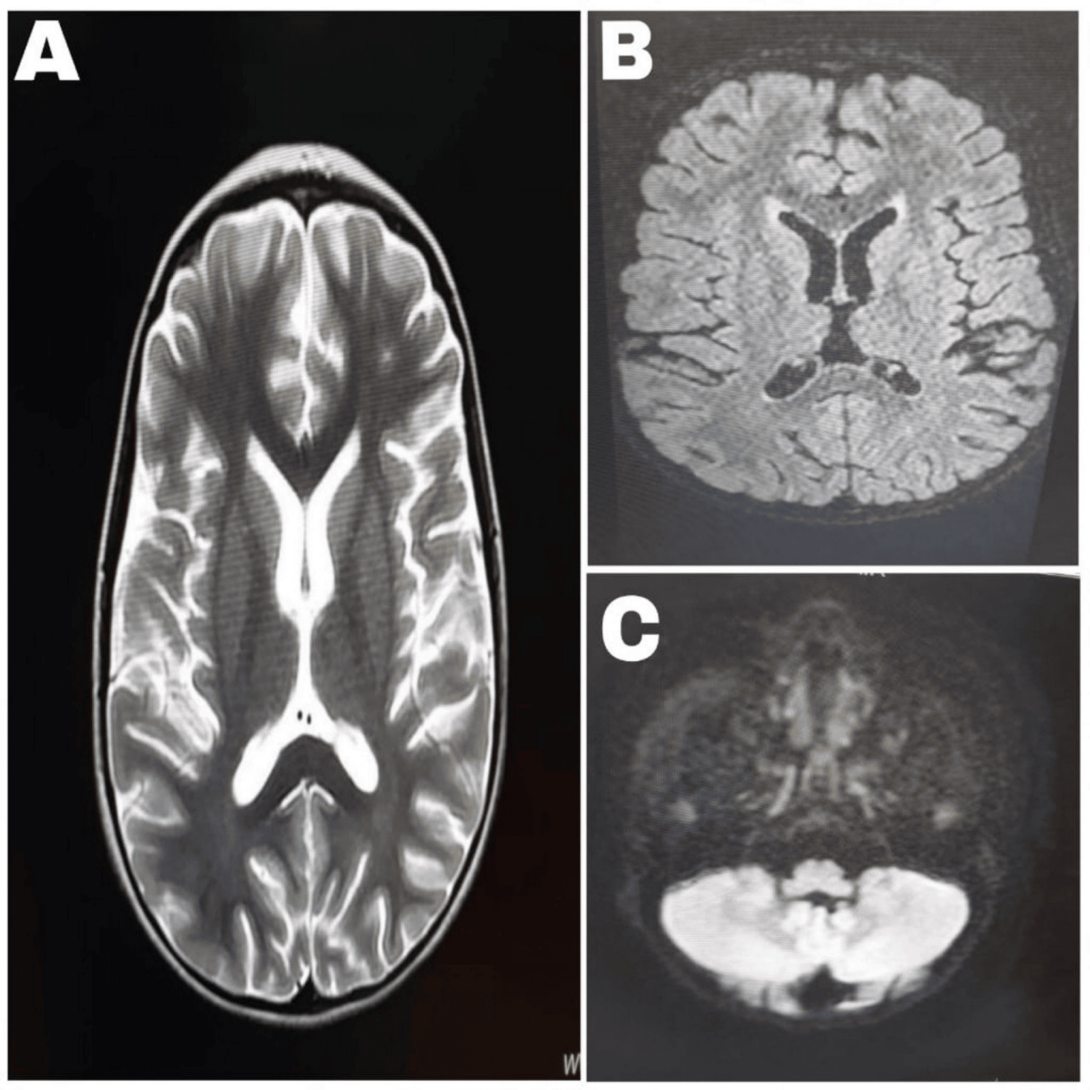
Follow-up brain magnetic resonance images showing complete lesion resolution. (A) Axial T2-weighted image showing disappearance of the splenial lesion. (B) Axial fluid-attenuated inversion recovery image showing complete resolution of the splenial abnormality. (C) Axial diffusion-weighted image showing complete resolution of the bilateral cerebellar lesions involving the dentate nuclei.

Clinical improvement became clear after intravenous immunoglobulin, with the return of smiling and social interaction on hospital day 15. She was able to stand with support on day 24 and regained verbal output and independent walking by day 28 after symptom onset. The total hospital stay was three weeks. A follow-up MRI performed one month after symptom onset showed complete resolution of the splenial and bilateral cerebellar lesions involving the dentate nuclei. At the three-month follow-up, she had complete neurological recovery with rehabilitation. She remained asymptomatic at two-year follow-up (Figures 2A-2C). The clinical course is summarized in Table 2.

**Table 2:** Timeline of clinical course and management. MERS: mild encephalopathy with a reversible splenial lesion

Time point	Event
November 2021	Presentation
Approximately day 30	Father had confirmed COVID-19, and several household members later developed compatible symptoms without testing
Day 7	Viral prodrome: fever, cough, rhinorrhea, myalgia, vomiting, headache
Hospital day 1	Brain CT scan normal, lumbar puncture performed on admission, multiplex CSF PCR panel negative; ceftriaxone and acyclovir started
Early hospitalization	SARS-CoV-2 serology: IgG positive, IgM negative
Hospital day 5	Brain MRI: splenial lesion and bilateral cerebellar lesions involving the dentate nuclei with diffusion restriction and no enhancement, MERS type II
Hospital day 6	Neurological worsening: loss of visual contact and dysphagia
Hospital days 7-9	Intravenous methylprednisolone 30 mg/kg/day for three days
Hospital days 12-13	Intravenous immunoglobulin 1 g/kg/day for two days, total dose 2 g/kg
Hospital day 15	Return of smiling and social interaction
Day 24 after symptom onset	Able to stand with support
Day 28 after symptom onset	Regained verbal output and independent walking
One month after symptom onset	Follow-up MRI: complete radiological resolution
Three months	Complete neurological recovery with rehabilitation
Two years	Asymptomatic

## Discussion

MERS is mainly described in children and is usually preceded by an infectious prodrome, with respiratory infections among the most frequent triggers [1,6]. Since the COVID-19 pandemic, MERS and related reversible splenial lesion syndromes have been reported to be associated with SARS-CoV-2 [3-5]. In our patient, household COVID-19 exposure, absence of SARS-CoV-2 vaccination, and positive SARS-CoV-2 immunoglobulin G (IgG) with negative immunoglobulin M (IgM) supported a probable post-infectious SARS-CoV-2 context, although the trigger was not microbiologically confirmed. In pediatric cohorts, type II disease is less frequent than type I [6,7], and extra-splenial lesions may involve the cerebellum, making this case notable for its bilateral dentate nucleus lesions and favorable long-term outcome [8,9].

The diagnosis of MERS is clinico-radiological and relies on the association of acute encephalopathic symptoms with a characteristic MRI pattern [1,2]. The typical MRI finding is a reversible splenial lesion, mildly hyperintense on T2-weighted and fluid-attenuated inversion recovery (FLAIR) sequences, with diffusion restriction and usually without contrast enhancement [1,2]. MRI also distinguishes type I, confined to the splenium, from type II, with lesions beyond it [6,7]. In our patient, brain MRI showed a splenial lesion and bilateral cerebellar lesions involving the dentate nuclei, both with diffusion restriction, consistent with MERS type II. A follow-up MRI at one month showed complete lesion resolution, further supporting the diagnosis, as lesion reversibility is a defining radiological feature of MERS. This characteristic imaging pattern also argued against alternative diagnoses such as acute disseminated encephalomyelitis and cerebellitis, while infectious meningoencephalitis was not supported by the unremarkable cerebrospinal fluid analysis, sterile bacterial culture, and negative multiplex cerebrospinal fluid polymerase chain reaction panel. Major metabolic causes were not supported by normal sodium levels, serum glucose, or renal function. Drug-related causes were unlikely because there was no exposure to or withdrawal of antiepileptic drugs and no use of implicated agents such as metronidazole or cyclosporine [2]. In this case, MRI was central to the diagnosis.

Management of MERS is primarily supportive and directed toward the neurological syndrome and its underlying cause. Most children recover completely, and the role of corticosteroids remains uncertain [6]. Empirical treatment was initiated because encephalitis could not be excluded at presentation. Guidelines recommend prompt intravenous acyclovir and empiric antibacterial therapy when clinically indicated [10]. In reported pediatric SARS-CoV-2-associated cases, management ranges from supportive care to immunomodulatory therapy in severe cases [3-5]. In our patient, neurological worsening with dysphagia led to treatment with methylprednisolone and then intravenous immunoglobulin. Improvement was observed after immunomodulatory therapy, although no causal conclusion can be drawn from a single case.

Prognosis is generally favorable, with most children showing complete clinical recovery and complete radiological resolution within days to weeks [1,6]. Type II disease may recover more slowly than type I [7]. This was also seen in our patient as follows: hypotonia, mutism, and gait improved gradually; and despite MRI normalization by one month, functional recovery was slower and required rehabilitation. Nonetheless, the outcome was excellent, with complete neurological recovery by three months and a normal course up to two years.

A limitation of this case is the absence of direct virologic confirmation of acute SARS-CoV-2 infection in the patient. Although an inflammatory post-infectious mechanism was considered, serum and cerebrospinal fluid cytokine profiling was not performed because these assays were not available in our setting; therefore, the correlation between inflammatory mediator levels and the clinical course could not be assessed. However, the epidemiological context, serological findings, characteristic MRI pattern, radiological resolution, and favorable long-term follow-up support the diagnosis of MERS type II in a probable post-infectious SARS-CoV-2 context.

## Conclusions

This case describes MERS type II in a six-year-old girl with reversible splenial and bilateral cerebellar lesions involving the dentate nuclei in a probable post-infectious SARS-CoV-2 context. Early recognition of this clinico-radiological pattern may facilitate diagnosis, help avoid unnecessary investigations and overly aggressive treatment, and support appropriate management.
